# Data on the nucleotide composition of the first codons encoding the complementary determining region 3 (CDR3) in immunoglobulin heavy chains

**DOI:** 10.1016/j.dib.2018.04.125

**Published:** 2018-05-04

**Authors:** Linnea Thörnqvist, Mats Ohlin

**Affiliations:** Dept. of Immunotechnology, Lund University, Lund, Sweden

## Abstract

The highly variable complementary determining region 3 (CDR3) of antibodies is generated through recombination of immunoglobulin heavy chain variable (IGHV), diversity, and joining genes. The codons encoding the first residues of CDR3 may be derived directly from the IGHV germline gene but they may also be generated as part of the rearrangement process. Data of the nucleotide composition of these codons of rearranged genes, an indicator of the degree of contribution of the IGHV gene to CDR3 diversity, are presented in this article. Analyzed data are presented for two unrelated sets of raw sequence data. The raw data sets consisted of sequences of antibody heavy chain-encoding transcripts of six allergic subjects (European Nucleotide Archive accession number PRJEB18926), and paired antibody heavy and light chain variable region-encoding transcripts of memory B cells of three subjects (European Nucleotide Archive accession numbers SRX709625, SRX709626, and SRX709627). The nucleotide compositions of the corresponding 5′-ends of sequences encoding the CDR3 are presented for transcripts with an origin in 47 different IGHV alleles. These data have been used (Thörnqvist and Ohlin, 2018) [1] to demonstrate the extent of incorporation of the 3′ most bases of IGHV germline genes into rearranged immunoglobulin encoding sequences, and the extent whereby any difference in incorporation affects the specificity of inference of the 3′-end of IGHV genes from immunoglobulin-encoding transcripts. They have also been used to assess the effect of observed gene differences on the composition of the ascending strand of CDR3 associated to antibodies with an origin in different IGHV genes (Thörnqvist and Ohlin, 2018) [1].

**Specifications Table**

**Table:** 

Subject area	Biology
More specific subject area	Immunobiology
Type of data	Figures, table
How data was acquired	Next generation sequencing (MiSeq, Illumina)
Data format	Analyzed
Experimental factors	Extraction of peripheral blood mononuclear cell RNA, construction of libraries encoding antibody heavy chain variable domains
Experimental features	Analysis of the nucleotide composition in the three most 5′ codons of the CDR3 of immunoglobulin heavy chain
Data source location	Lund, Sweden
Data accessibility	Analyzed data are available within this article. Raw data generated by us [2], [3] are available in the European Nucleotide Archive, with accession number PRJEB18926 (www.ebi.ac.uk/ena/data/view/PRJEB18926). Additional raw data [4] also analyzed as part of this study are available from the European Nucleotide Archive (accession numbers SRX709625, SRX709626 and SRX709627).

**Value of the data**

• These data are useful for further development of processes used to infer the immunoglobulin gene repertoire of an individual, and for interpretation of the results of such analyses.
• These data are useful for further development of processes used to infer new germline gene sequences.
• These data are useful to investigators of antibody repertoire as they suggest avenues to identify the existence of, to this date, unrecognized alleles of immunoglobulin germline genes.
• These data are useful for interpretation of sequence diversity in the ascending strand of CDR3 of naïve and antigen-specific immune repertoires.

## 1 Data

**Fig. 1:**
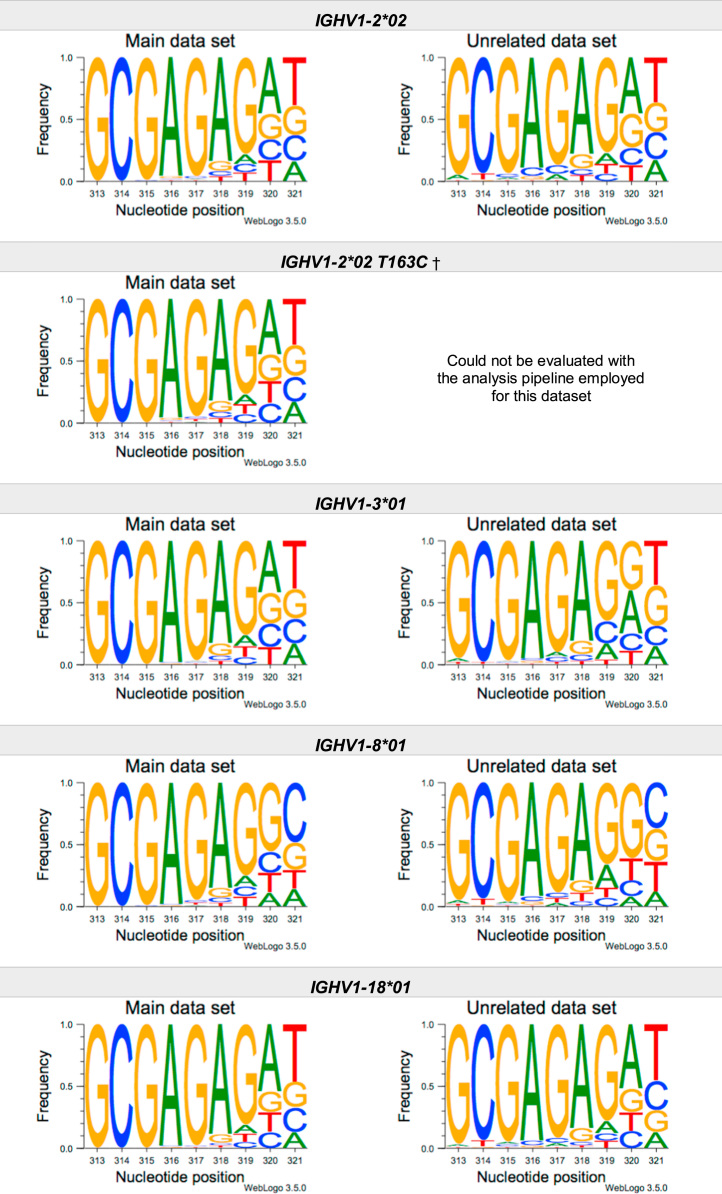

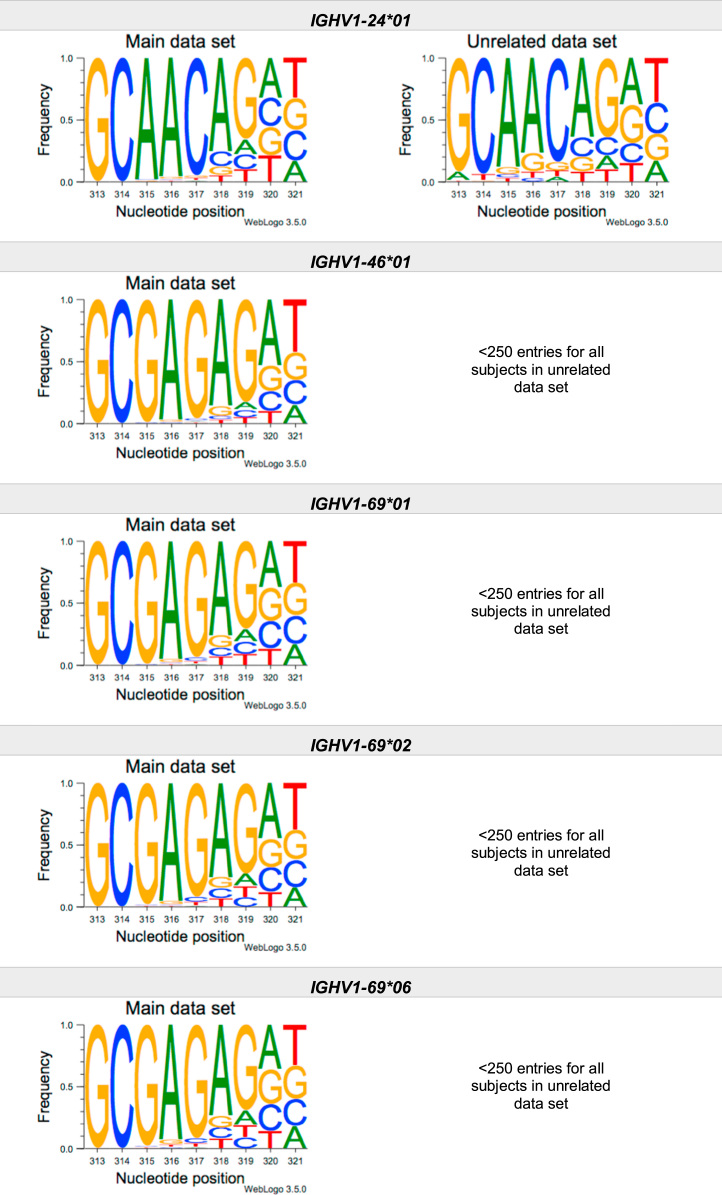

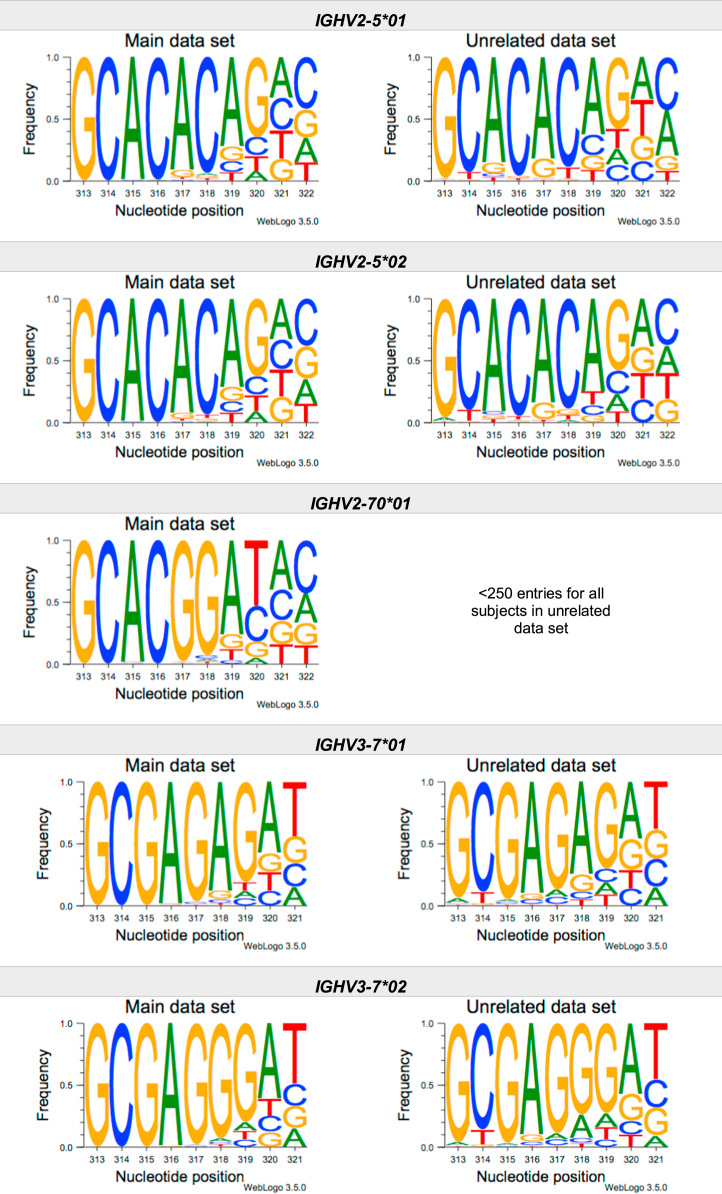

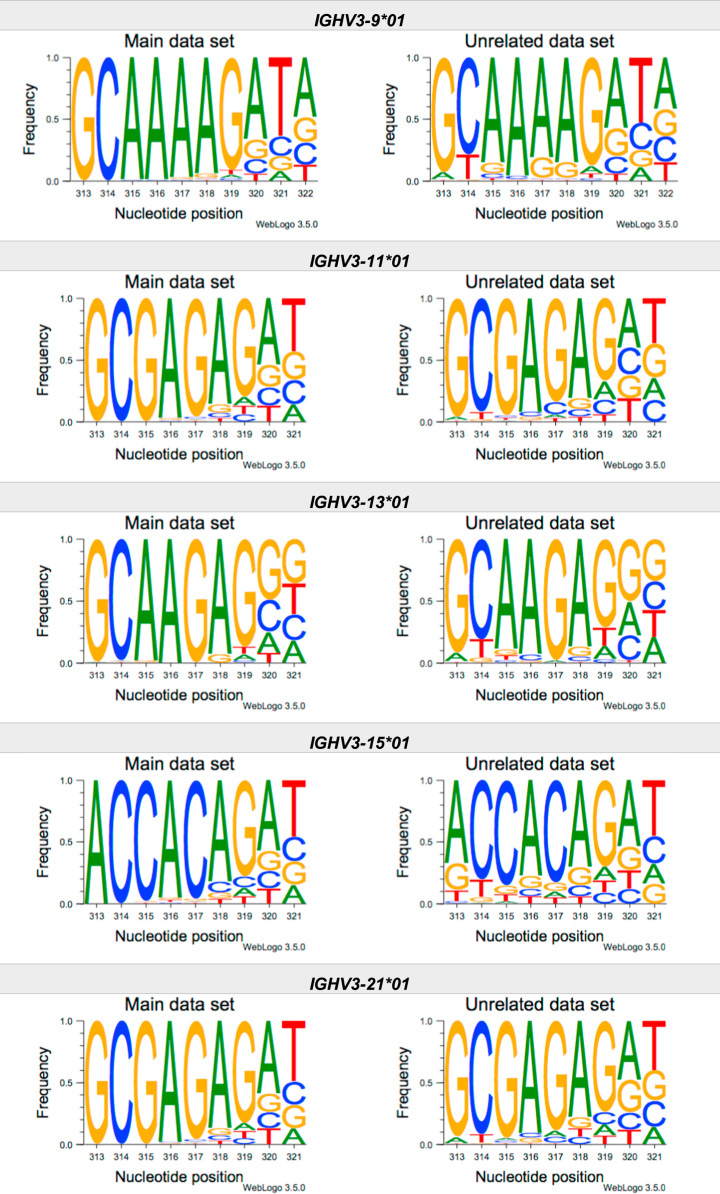

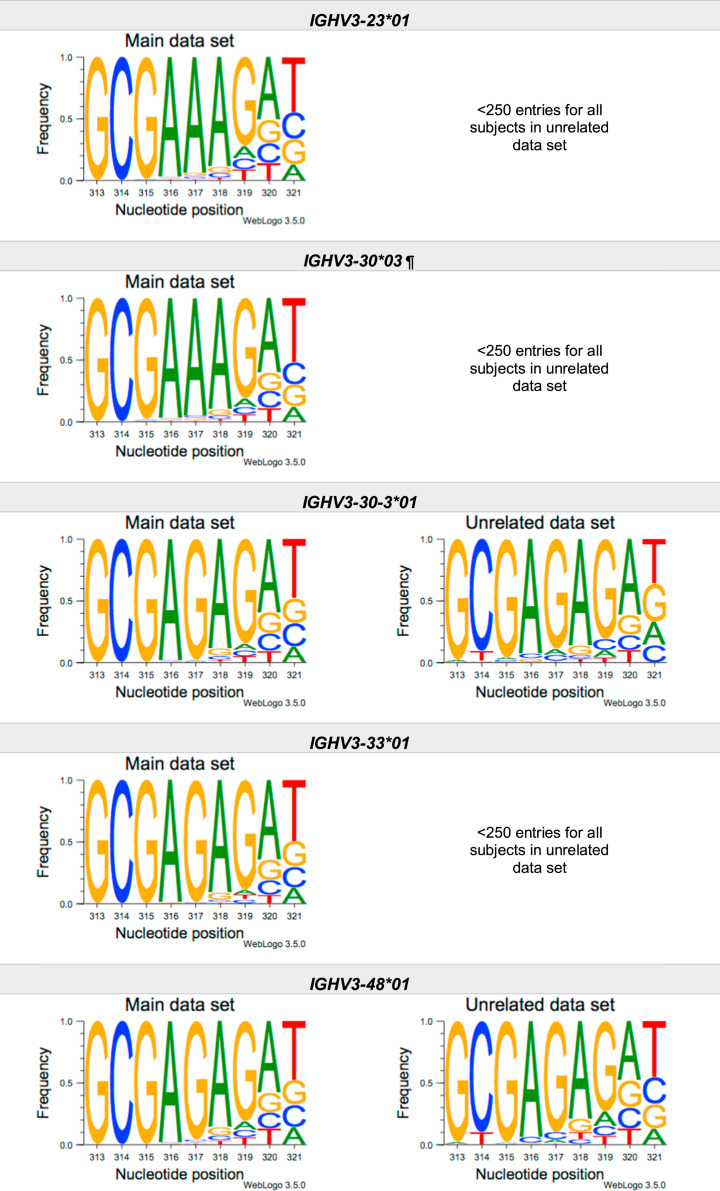

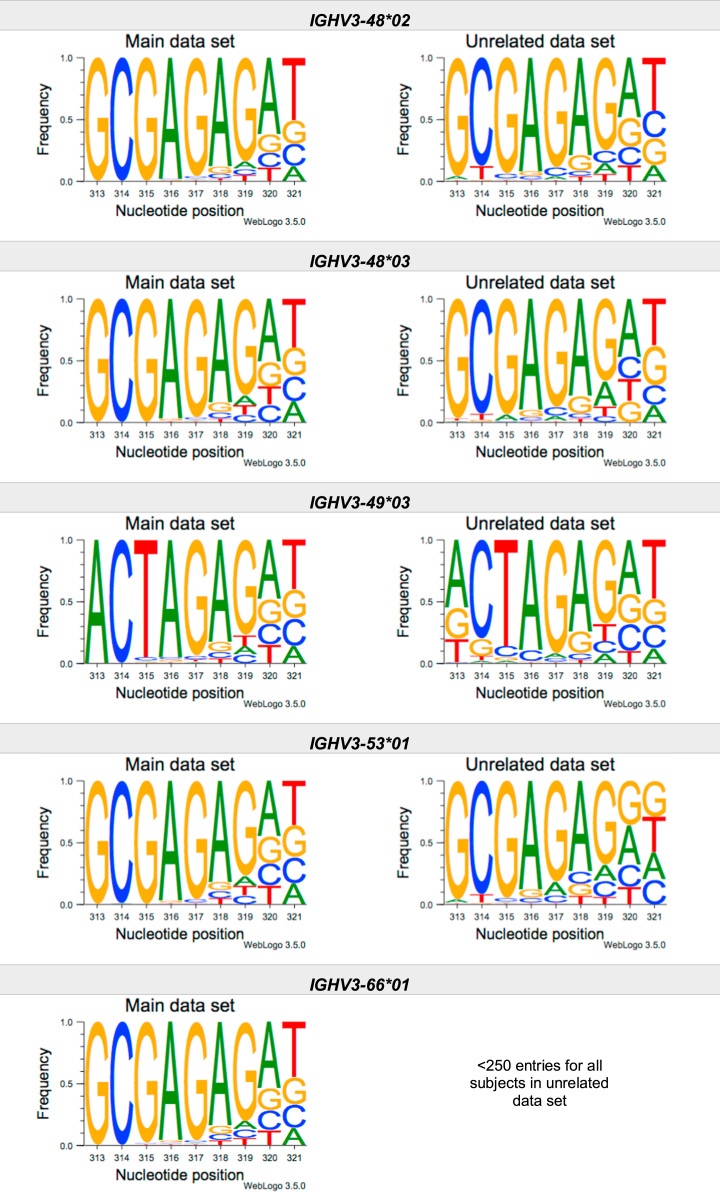

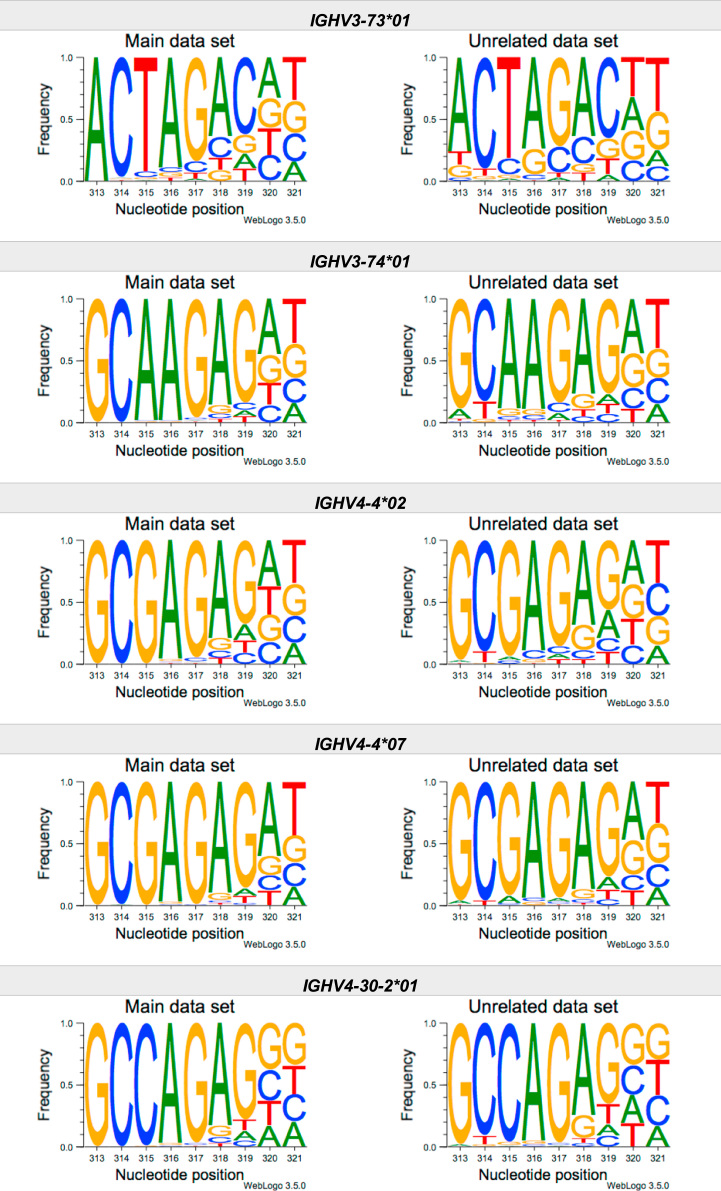

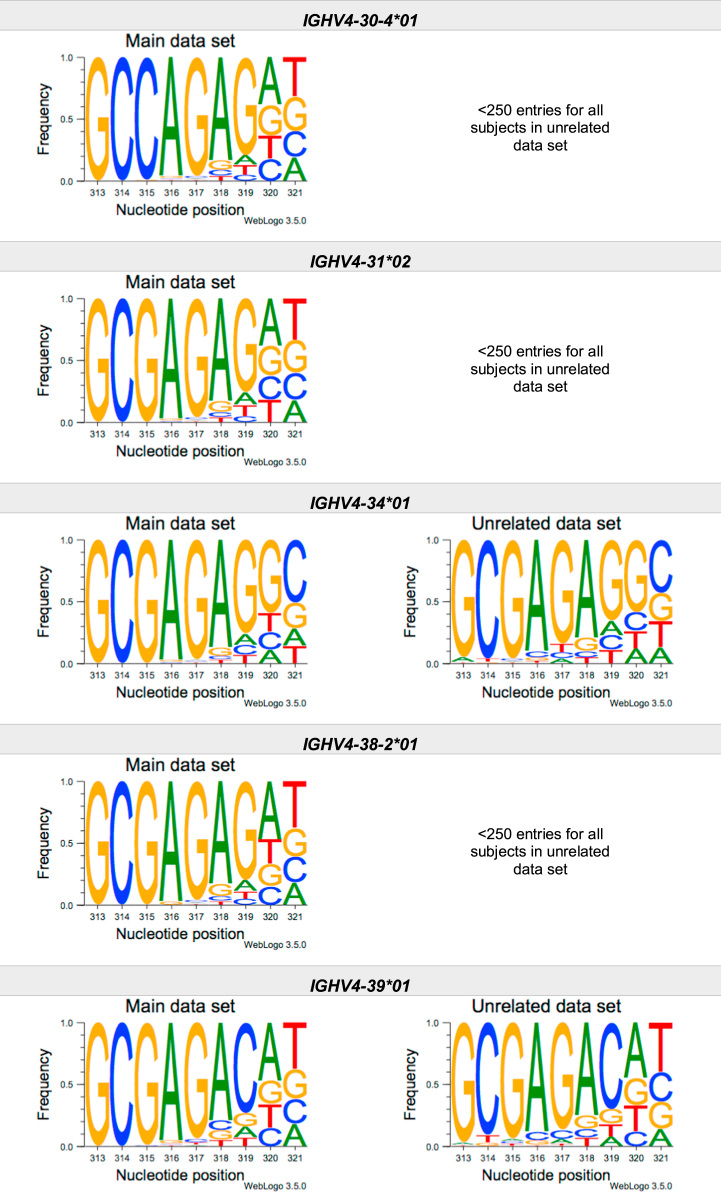

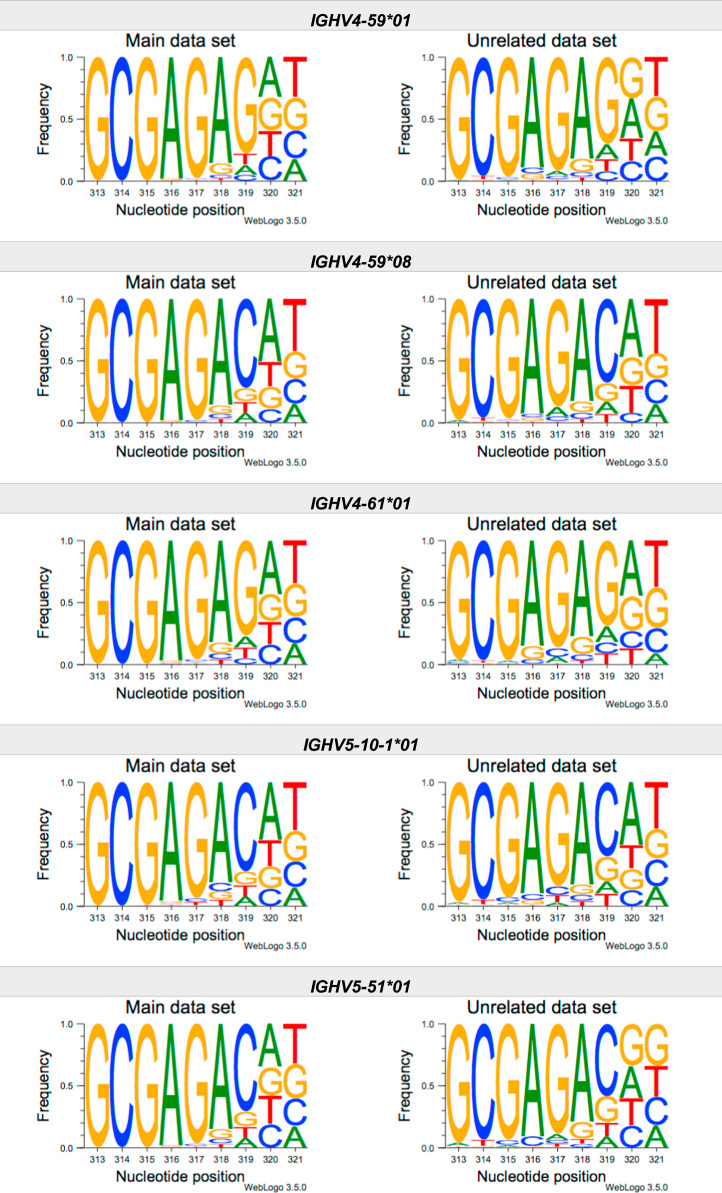

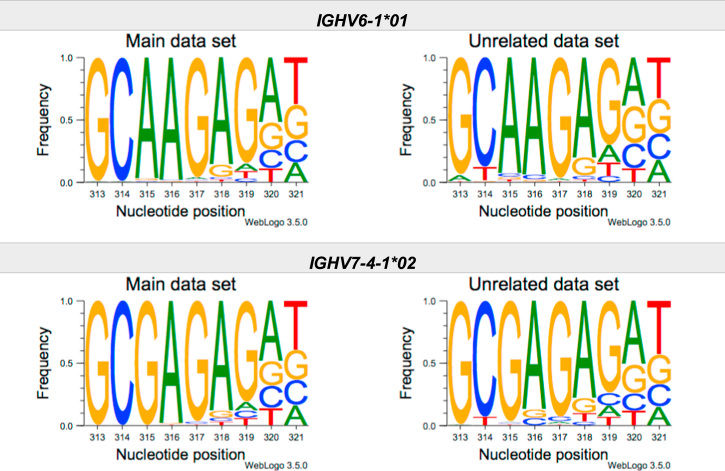
Distribution of bases in the first three codons of 47 genes/alleles encoding CDR3 of antibody heavy chains in the main examined data set [2], [3] and in an unrelated data set [4]. For the latter data set, only transcripts that were exclusively inferred to one germline gene/allele were used. IGHV1–2*02 T163C (†) would be inferred as either IGHV1–2*02 or IGHV1–2*05, and could thus not be evaluated with the used method. IGHV3–30*03 (¶) and IGHV3–30*18 are identical in the part of the sequence that is inferred by the used approach, but differ in codon 106 where they carry an *AGA* and an *AAA* trimer, respectively. Hence, transcripts that herein have been inferred as derived from IGHV3–30*03 more likely originates from IGHV3–30*18, since they predominantly incorporated an *AAA* trimer in codon 106. The number of subjects used for analysis varies between 3 and 6 in the main data set and 0 and 3 in the unrelated data set (Table 1).

**Fig. 2:**
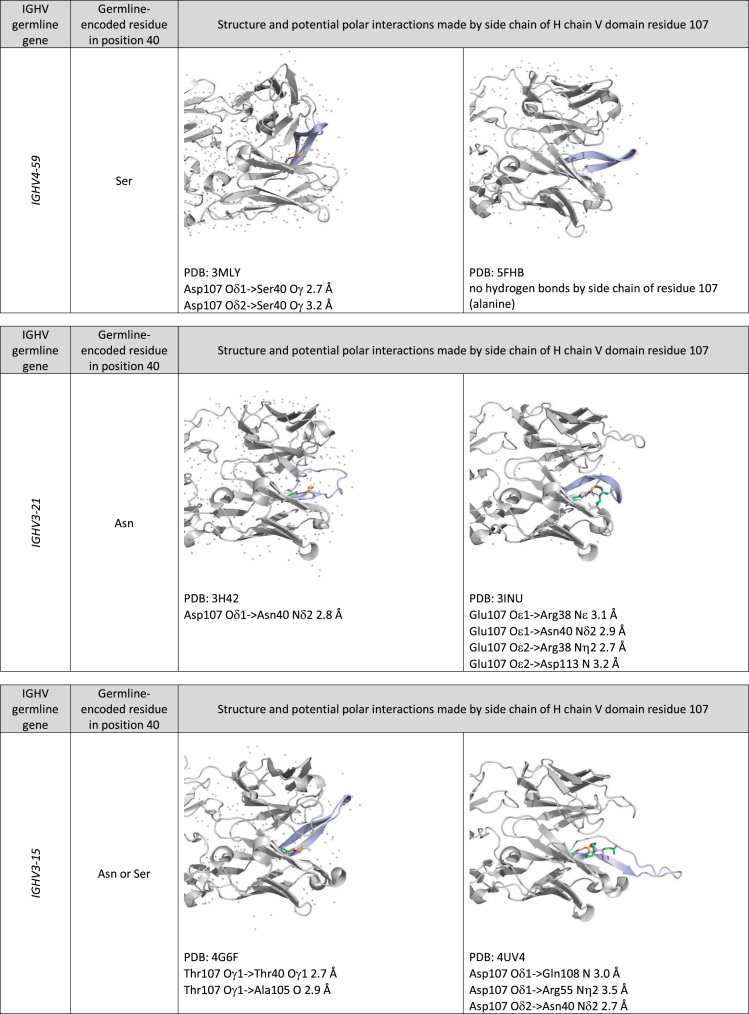

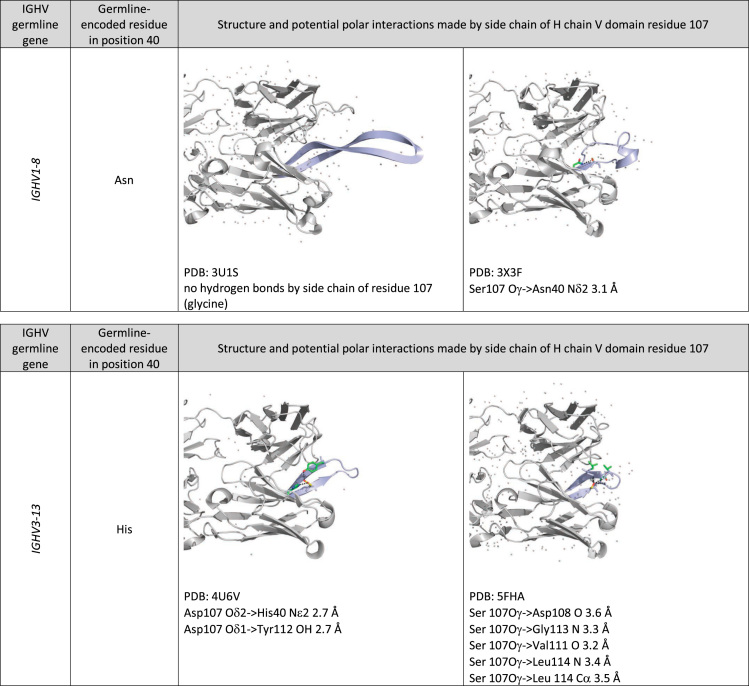
Examples of position of and potential polar interactions made by the side chain of H chain V domain residue 107. Carbon atoms of the side chain of residue 107 are highlighted in yellow and those of the side chain of other residues are highlighted in green. The backbone of H chain CDR3 is shown in light blue.

This article present data of nucleotide composition in antibody heavy transcripts originating in 47 different immunoglobulin heavy chain variable (IGHV) germline genes/alleles (Fig. 1) [1]. The data is limited to the three most 5′ codons (codon 105–107, according to IMGT numbering [5]) that encode the sequence of the complementary determining region 3 (CDR3). For transcripts originating in germline genes that encodes also the first base of the fourth codon of CDR3 (codon 108), the nucleotide composition at this position is also presented. The location of, and polar interactions potentially mediated by, the side chain of amino acid residue 107 in a set of antibody structures is shown (Fig. 2). The number of subjects that contributed sequence information for the generation of Fig. 1 is summarized in Table 1.

**Table 1:** Number of subjects in which the number of transcript entries exceeded the cut-off value.

	Main Data Set	Unrelated Data Set
	Number of subjects with > 500 entries	Number of subjects with > 250 entries
IGHV1–2*02	5	3
IGHV1–2*02 T163C	3	Not evaluated
IGHV1–3*01	4	2
IGHV1–8*01	6	2
IGHV1–18*01	6	3
IGHV1–24*01	4	3
IGHV1–46*01	6	0
IGHV1–69*01	6	0
IGHV1–69*02	3	0
IGHV1–69*06	3	0
IGHV2–5*01	3	1
IGHV2–5*02	6	1
IGHV2–70*01	3	0
IGHV3–7*01	6	3
IGHV3–7*02	3	2
IGHV3–9*01	6	3
IGHV3–11*01	6	2
IGHV3–13*01	3	1
IGHV3–15*01	6	3
IGHV3–21*01	6	3
IGHV3–23*01	6	0
IGHV3–30*03	6	0
IGHV3–30-3*01	5	1
IGHV3–33*01	6	0
IGHV3–48*01	4	2
IGHV3–48*02	4	2
IGHV3–48*03	3	1
IGHV3–49*03	5	2
IGHV3–53*01	5	2
IGHV3–66*01	3	0
IGHV3–73*01	3	1
IGHV3–74*01	6	3
IGHV4-4*02	6	3
IGHV4-4*07	4	2
IGHV4–30-2*01	4	2
IGHV4–30-4*01	6	0
IGHV4–31*02	6	0
IGHV4–34*01	6	3
IGHV4–38-2*01	3	0
IGHV4–39*01	5	3
IGHV4–59*01	6	3
IGHV4–59*08	3	1
IGHV4–61*01	6	2
IGHV5–10-1*01	3	1
IGHV5–51*01	6	2
IGHV6-1*01	5	2
IGHV7-4-1*02	3	1

The cut-off value was set to 500 entries for the main data set [2], [3] and to 250 entries for the unrelated data set [4]. For the latter, only transcripts that were exclusively inferred to a single germline allele were used.

## 2 Experimental design, materials and methods

### 2.1 Sample collection, library construction and sequencing

Peripheral blood and bone marrow samples of six allergic subjects were collected (approved by the regional ethical review board at Lund University), and used to construct libraries of antibody H chain V domains, as previously described [2]. In brief, isolated mononuclear cells where divided into duplicate samples from which RNA was extracted. Subsequently, cDNA was produced from the RNA and amplified with Biomed2 primers [6] targeting sequences encoding the constant domain (isotype-specifically) and the first framework region of antibody H chains, respectively. The products were barcoded and subsequently sequenced at National Genomics Infrastructure (SciLifeLab, Stockholm, Sweden), using MiSeq technology (Illumina, Inc. San Diego, CA, USA) and a paired-end setting (2 × 300 bp) [2].

### 2.2 Processing of sequencing data

FASTQ raw data files (available at the European Nucleotide Archive with accession number PRJEB18926) generated in our laboratory, constituted the main data set. They were processed as previously described [2]. The sequences were filtered, trimmed, paired, assembled and divided in isotype specific FASTA files using pRESTO 0.4.4 [7], and the isotype annotation were confirmed through evaluation of the presence of isotype-specific sequences. Any sequences lacking such were discarded [2]. Germline genes were inferred for IgM encoding sequences using IgDiscover [8], as previously described [9]. Germline gene libraries retrieved from IMGT [10] were used, but with the IGHV library adjusted to cover no more than codon 25–105. Finally, sequences were filtered so that only those that encoded at least eight amino acids in the CDR3, that covered at least 99% of the inferred IGHV germline gene and that lacked errors compared with the inferred IGHV gene were further analysed.

Another, unrelated set of raw sequence data was downloaded from the European Nucleotide Archive (accession numbers SRX709625, SRX709626 and SRX709627) [4], and prepared for analysis. The data set contained transcripts from peripheral blood memory B cells encoding paired H chain V domain and light chain V domain in three subjects, and were generally processed as described above, but using pRESTO 0.5.4 [7]. As the isotype encoded by the transcripts was unknown, no dividing of sequences with regard to isotype were performed. Consequently, IgDiscover [8], which mostly are designed for IgM analysis, could not be used for germline genes inference. Instead, duplicate sequences were removed using the pRESTO 0.5.4 CollapseSeq tool [7] and IGHV gene were subsequently inferred using IMGT HighV-QUEST [11]. For further analysis, only sequences inferred as productive to one single allele of an IGHV gene and that had at least eight amino acids in the CDR3 were used.

### 2.3 Analysis of nucleotide composition in CDR3 codons encoded by IGHV germline gene

The nucleotide composition of the first three codons of the CDR3 region, which are encoded by the IGHV gene, were analysed for each donor of both the main and the unrelated data set. In total, transcripts originating in 47 different alleles of IGHV genes were studied, each of them having at least 500 transcripts in at least three of the donors of the main data set. Mean frequency of nucleotide bases at each examined position were calculated for both data sets separately. For the main data set, only values from subjects with at least 500 transcripts originating in a certain allele of an IGHV gene were considered. For the unrelated data set, this limit was set to 250 transcripts. The number of subjects for which these conditions were fulfilled is summarized for each allele in Table 1. The mean frequency values were used to construct the illustrations presented in Fig. 1, using WebLogo 3.5.0 [12].

Most of the studied IGHV genes may contribute to nucleotides of the first three codons that encode the CDR3 (codon 105–107, as defined by the IMGT numbering system [5]). Hence, these are the codons for which the nucleotide composition generally was analysed. Four of the germline genes/alleles (IGHV2–5*01, IGHV2–5*02, IGHV2–70*01, and IGHV3–9*01) may however also encode the first base of codon 108. Thereby, the nucleotide composition was analysed also at this position for transcripts originating in any of these four germline genes/alleles.

### 2.4 Protein structures

Example structures of antibodies encoded by genes with a particular germline gene origin were identified using IMGT/3Dstructure-DB [13]. Protein structure coordinates were downloaded from the Protein Data Bank (https://www.rcsb.org). The structures were visualized using MacPyMol 1.8.0.6 (The PyMOL Molecular Graphics System, Schrödinger, LLC).
