# Supplementary material for: Data on the nucleotide composition of the first codons encoding the complementary determining region 3 (CDR3) in immunoglobulin heavy chains
Source: Data Brief. 2018 May 4;19:337–52. doi: 10.1016/j.dib.2018.04.125 (PMC5992955; doi:10.1016/j.dib.2018.04.125)
Supplement: Supplementary file 1 — Supporting information [file mmc1.docx]

**Declarations of interest**

All the author confirms that there are no Conflict of interest.
